# A mathematical model of the impact of present and future malaria vaccines

**DOI:** 10.1186/1475-2875-12-126

**Published:** 2013-04-15

**Authors:** Edward A Wenger, Philip A Eckhoff

**Affiliations:** 1 Intellectual Ventures Laboratory, , 1555 132nd Ave NE, Bellevue, WA 98005, USA

## Abstract

**Background:**

With the encouraging advent of new malaria vaccine candidates, mathematical modelling of expected impacts of present and future vaccines as part of multi-intervention strategies is especially relevant.

**Methods:**

The impact of potential malaria vaccines is presented utilizing the EMOD model, a comprehensive model of the vector life cycle coupled to a detailed mechanistic representation of intra-host parasite and immune dynamics. Values of baseline transmission and vector feeding behaviour parameters are identified, for which local elimination is enabled by layering pre-erythrocytic vaccines of various efficacies on top of high and sustained insecticide-treated net coverage. The expected reduction in clinical cases is further explored in a scenario that targets children by adding a pre-erythrocytic vaccine to the EPI programme for newborns.

**Results:**

At high transmission, there is a minimal reduction in clinical disease cases, as the time to infection is only slightly delayed. At lower transmission, there is an accelerating community-level protection that has subtle dependences on heterogeneities in vector behaviour, ecology, and intervention coverage. At very low transmission, the trend reverses as many children are vaccinated to prevent few cases.

**Conclusions:**

The maximum-impact setting is one in which the impact of increasing bed net coverage has saturated, vector feeding is primarily outdoors, and transmission is just above the threshold where small perturbations from a vaccine intervention result in large community benefits.

## Background

In recent years, dramatic progress has been made in reducing the burden of malaria through the scale-up of insecticide-treated net (ITN) coverage and the increasing use of artemisinin combination therapy (ACT) as first-line treatment [1-4]. Nonetheless, as of the 2010 WHO report there were still over 200 million cases of malaria per year with 800 thousand attributed deaths, mostly young sub-Saharan African children [1]. Further reductions in burden and large-scale clearance of the disease will increasingly depend on combinations of interventions. In the face of potential drug resistance [5,6], insecticide resistance [7], and vector behaviour changes [8,9] an effective vaccine could become an increasingly important tool.

Malaria vaccine development is particularly challenging given the complexity of the parasite life cycle and the variation in its antigenic presentation [10-12]. In spite of the early potential demonstrated in studies of irradiated sporozoites [13], it has only been in the last few years that vaccine trials have shown sufficiently high and durable efficacies to consider widespread deployment. The RTS,S subunit vaccine targeting the circumsporozoite protein (CSP) in the pre-erythrocytic phase has advanced the furthest along the clinical trial pathway among current vaccine candidates. In Phase IIb and Phase III field trials to date, the following protective efficacies have been observed: 34% against infection in Gambian adults waning rapidly over 15 weeks [14]; 30% against clinical episodes and 45% against infection in Mozambican children over 6 months [15] and persisting at similar levels out to 21 months [16]; 49–56% against clinical episodes over a year in children from Kenyan and Tanzanian children [17] and from seven African countries [18,19]. These trials are consistent with a ‘leaky’ vaccine that provides partial protection to most vaccinated individuals [20,21] by reducing the number of successful sporozoites and the size of the liver-to-blood inoculum [22]. Beyond RTS,S, there are additional pre-erythrocytic vaccine candidates, as well as blood-stage vaccines to reduce morbidity and gametocyte-blocking vaccines to limit human-to-vector transmission [23]. While this latter class of ‘sexual-stage’ vaccines does not provide direct individual protection against infection, it may be a potent tool in bringing down malaria transmission. Recent Phase I trials targeting the sexual stage of the parasite, in particular the ookinete surface proteins Pfs25 and Pfs28, have demonstrated persistent high antibody levels that are effective in blocking oocyst formation in the mosquito [23-26].

The objective of the present modelling study is to quantify the potential impact of first-generation vaccine candidates as components in multi-intervention elimination strategies, to elucidate which geographical settings in a wide range of transmission intensities and entomological behaviours enable the greatest impact, and to estimate efficacy targets for future vaccines as a function of desired impact. Among the many malaria vaccine scenarios to which mathematical modelling may be applied, the focus of the present effort is limited to simulating pre-erythrocytic and sexual-stage vaccines, which are distributed either by mass campaign or by routine vaccination. Their effects will be quantified in terms or reduced inoculation rates, prevalence, clinical incidents, and interruption of transmission. Mathematical modelling is especially relevant today as some countries are looking for evaluations to inform decisions and potential plans regarding incorporating RTS,S into their national Expanded Programme on Immunization (EPI).

The present work builds upon a substantial body of work by the malaria modelling community to understand the potential impact of malaria vaccines. Early malaria models from Ross and Macdonald [27,28] were used to focus on vector control. The modelling work to support The Garki Project [29] represented a major advance in modelling capabilities; evolutions of this model were used in early models of vaccine action [30]. The clinical trials of RTS,S and its potential rollout have helped to drive forward a new generation of mathematical models with new features, new capabilities, and more detailed representations of parasites and immunity [31-35]. The community is benefiting from the array of independent modelling efforts to understand vaccine rollouts, and more benefits will accrue as ensemble modelling becomes the standard in the field [36].

## Methods

The present study utilizes the Disease Transmission Kernel (DTK) model developed by the Epidemiological Modelling (EMOD) group at Intellectual Ventures. This individual-based model couples a detailed description of the vector life cycle [37] with a comprehensive, mechanistic representation of the intra-host parasite and immune dynamics [38]. The model additionally includes a flexible and powerful framework for configuring and distributing arbitrarily specified campaign interventions to targeted groups of individuals [39].

### Vector model

Multiple species of *Anopheles* mosquitoes may be simulated simultaneously, each population separately configured according to its ecological and behavioural preferences. For example, the female *Anophelesarabiensis* deposits eggs primarily in temporary, rainfall-driven habitat and has a higher propensity to feed outdoors [8] or on livestock [40]. Vector populations are tracked as cohorts throughout the full mosquito life cycle: from eggs to larvae with a temperature-dependent development period preceding emergence; a brief immature phase including sugar-feeding and mating; repeating multi-day gonotrophic cycles during which mosquitoes may be exposed to infection by gametocytes; and a temperature-dependent latency for sporogony [41]. This closed-loop feeding cycle ensures that only successful blood meals, which avoid potentially multiple modes of vector control, result in viable eggs.

Critical to modelling multiple simultaneous interventions accurately, the various feeding cycle outcomes – death before, during, or after feeding; host unavailable; successful human feed; etc. – are calculated from branching trees of conditional probabilities, the nodes of which can be influenced by individual interventions. For example, in the case of indoor-host-seeking mosquitoes encountering both indoor residual spraying (IRS) and an ITN, feeding outcomes will branch first at pre-feed IRS killing and repellency from the house. The fraction surviving these fates can be blocked by the ITN, which may additionally kill a subset of the blocked fraction. The unblocked fraction makes a feeding attempt, during which some may be killed. Those surviving the feeding attempt may at last be killed by IRS post-feed. Thus, the deterrent and toxic effects of multiple interventions can be represented simultaneously. The allocation of mosquitoes to feeding-cycle outcomes is done based on end-state probabilities that have been aggregated over the individual humans in the simulation. The presented simulations do not examine interventions targeting other phases of the vector life cycle (e.g. larvicides, larval habitat management) or those targeting outdoor or animal feeds, as these are not common components in vaccine rollout scenarios. Nonetheless, the vector transmission model supports such interventions.

### Malaria infection and immune model

Within-host parasite dynamics are simulated by a microsolver for each individual in the simulation. Within the framework of this microsolver, each new *Plasmodiumfalciparum* infection begins with a fixed-duration, liver-stage latency that is susceptible to drugs such as primaquine. The latent period ends with a configurable burst of merozoites that commences the repeating two-day cycles of the asexual blood-phase infection. The model accounts for several antigenic components, to which the immune system may develop immunity: the merozoite surface protein (MSP) variant, the *P.falciparum* erythrocyte membrane protein (PfEMP-1) presented on the surface of the infected red-blood cell (IRBC), and less immunogenic minor surface epitopes. A single clonal infection is modelled with an antigenic repertoire of 50 unique PfEMP-1 variants, each associated with one of five repeating minor epitopes. In the first asexual cycle, the first five variants are expressed in equal numbers. The blood-stage infection is updated in hourly time steps, during which the immune system is stimulated by the IRBC count of each antigenic variant, and concomitantly the IRBC counts are decremented on account of immune and drug killing effects.

At the end of each asexual cycle, the model calculates the fraction of merozoites (16 for each previous IRBC) that are killed by specific recognition of the MSP variant. It also calculates the fraction that is differentiated into male and female gametocytes. To capture the dynamics of the parasite’s immune evasion strategy, the model imposes a constant per-parasite switching rate on the remaining merozoites for advancing to subsequent antigenic variants in the repertoire. Super-infection is allowed for up to five simultaneous infections. The full set of population-level antigenic variants, out of which a single infection’s repertoire is randomly drawn, consists of 100 MSP variants, 20 sets of five minor epitopes, and 1000 PfEMP-1 variants. The number of population-level variants affects the age-pattern of natural immunity acquisition. The former two parameters drive the asymptotic levels of adult detected parasitaemia; the latter parameter primarily governs the transition between child and adult detected prevalence rates, provided the number of PfEMP-1 variants is substantially more than an individual would experience in a year. As in a previous study on the acquisition of immunity through the mechanism of parasite population diversity [42], the numbers of antigenic variants in this study were chosen to resemble age-prevalence curves from Namawala, Tanzania [43]. A ‘burn-in’ period allows simulated individuals to build antibody responses to a broad repertoire of parasite antigens appropriate for their age, before perturbing the system with campaign interventions. The simulations presented here used a ten-year burn-in – a trade-off that allows population-level transmission to approach asymptotic dynamics while making appropriate use of available computing resources.

The immune response to infection is characterized by innate inflammatory and specific antibody components. The cytokine-driven inflammatory response is modelled to depend on a temporary contribution from rupturing schizonts at the end of each asexual cycle, as well as the concentration of IRBC surface antigens to which an antibody response has not yet been developed. The innate response – suppressed by the presence of specific antibodies – is responsible for driving febrile symptoms and broad-spectrum parasite suppression. The capacity to generate specific antibodies grows in response to the concentration of each novel antigen. Above a threshold level on the capacity, antibodies are produced in increasing concentration until the corresponding antigenic variant is cleared, at which time the capacity will decay to a non-zero memory level. This mechanism captures the delayed onset of specific antibody response that is reduced on re-infection.

The model advances gametocytes through five stages of development, characterized by different drug susceptibilities, reaching maturity after ten days. Infectiousness of individuals to mosquitoes is proportional to the number of mature gametocytes taken up in a blood meal, but modulated by a factor that inactivates gametocytes at high cytokine densities [44-49]. Peak infectiousness typically follows peak parasitaemia by about ten days. The contribution of different age groups to transmission is dependent on the more pronounced innate inflammatory response in malaria-naïve individuals, as well as the different durations of infection. The transmission-blocking effect of sexual-stage vaccines is also applied at this stage to directly reduce the probability of a viable human-to-vector infection.

In the present study, the probability of a sporozoite-positive mosquito feed infecting an individual is based on a single random draw with 50% average transmission rate [50]. In the model, the generic pre-erythrocytic vaccine takes an input efficacy parameter that defines the fractional reduction in the probability of an infectious bite causing an infection. In a time step (*Δt*) with a daily infectious biting rate (*R*), the probability of an individual with vaccine efficacy (*E*) acquiring a new infection is: 1−*exp*(−*R*·(1−*E*)·*Δt*). For the sake of simplicity, several additional model features related to vector-to-human transmission have not been exercised in this analysis. These include the potential to assign heterogeneous susceptibility, biting rates, and CSP-specific antibody production in response to repeated challenges.

### Simulated population and geography

Simulations have been run on the scale of a single village with an initial population of 1,000 individuals, distributed exponentially in age with a mean of 23.2 years. The annual birth rate of 36.5 per 1,000 individuals and the age-dependent non-disease mortality rate result in a population doubling time of roughly 20 years. The age structure of individual immune systems is initialized through a 10-year ‘burn-in’ period, after which various interventions – insecticide-treated bed nets, pre-erythrocytic vaccines (PEV), and sexual-stage vaccines – are distributed to a subset of the population.

The climate and vector parameters were modelled on the well-instrumented study site of Namawala, Tanzania [51]. The rainfall data for January 1990 through December 1999 were obtained from the Global Precipitation Climatology Centre (GPCC) [52], and the temperature data from the Swiss Tropical and Public Health Institute (TPH) [31]. The simulation includes distinct populations for each of the three dominant species: *Anophelesgambiaes.s.*, *Anophelesfunestus*, and *An.arabiensis*, which account for around 10%, 20%, and 70% of annual infectious bites, respectively. The scale factors governing the available habitat for each species have been set as in [37] by fitting basic vector-model simulations with the seasonality and species-mix of the Namawala series; the overall magnitude is varied over a range of values of the entomological inoculation rate (EIR) that are all below those historically seen in Namawala. The baseline transmission has a pronounced rainy season, during which individuals in the high-transmission scenarios average more than one infectious bite per day. During the dry season, though, the *An.funestus* component plays a disproportionately significant role, when for several months it accounts for the majority of infectious bites. In order to present results on vaccine impact that are more broadly applicable to diverse geographic settings, simulations were configured over a range of values for the annual EIR (0 - 150) and the fraction of *An.arabiensis* feeds that occur indoors (0 - 1).

## Results and discussion

The simulated outputs presented in this paper address two distinct deployment scenarios. The first section explores the effect of adding a pre-erythrocytic or sexual-stage mass-vaccination event to a large-scale distribution of ITNs. This is not necessarily how first-generation vaccines are initially conceived to be deployed, but it is an instructive exercise to understand what can or cannot be achieved towards a goal of local disease elimination with these tools alone. This scenario also explores the potential further reduction in burden once ITN campaigns have saturated in their possible local impact. The second section explores the effect of an EPI-like deployment of pre-erythrocytic vaccines on the number of clinical disease cases in various age groups. These results should be directly relevant to current and future vaccine deployment plans, especially in pointing out the settings where the greatest impact may be realized.

### Mass-vaccination towards eradication

**Figure 1:**
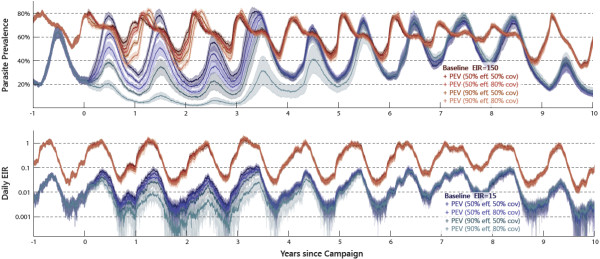
**Effect of mass PEV distribution on parasite prevalence and EIR.** The fraction of individuals with slide-microscopy detectable parasitaemia (upper panel) and the daily entomological inoculation rate (lower panel) for ‘baseline’ simulations (darkest lines) in addition to four PEV-distribution scenarios: 50% (red and blue lines) and 90% (orange and teal lines) efficacy, each at 50% or 80% demographic coverage. Results are shown both for high-intensity (EIR=150, redder hues) and medium-intensity (EIR=15, bluer hues) transmission. The mean and standard deviation of 10 stochastic simulations are shown as lines and shaded areas, respectively. The *An.arabiensis* indoor feeding fraction is 50% at both transmission intensities.

Figure 1 shows the simulated average population prevalence as detectable by slide microscopy (detection threshold: 10 IRBC per *μ*L) and the daily EIR in the decade following a single mass-vaccination event with a PEV efficacy that decays exponentially (5-year decay constant) from the initially specified value. Each trace is the mean of 10 stochastic simulations, with the 1-sigma variance represented by shaded bands. Redder colours correspond to higher pre-intervention baseline transmission (EIR=150), while bluer colours represent lower transmission (EIR=15). Five scenarios of increasing impact are shown for each transmission level: a baseline scenario; 50% initial PEV efficacy and 50% demographic coverage; 50% efficacy and 80% coverage; 90% efficacy and 50% coverage; and finally, 90% efficacy and 80% coverage. At lower transmission and with 50% indoor *An.arabiensis* feeding, the potential impact can be substantial. In contrast, the high-transmission scenario has sufficiently high biting rates (even during the dry season) that seasonal oscillations in prevalence are less prominent and there is minimal impact due to the mass-vaccination. Slightly more individuals recover at the beginning of the dry season, and the rise with the oncoming wet season is postponed. Note that the last year of these simulations had very low rainfall in the Namawala climate series, and thus the parasite levels are lower than the preceding years, especially at lower transmission intensities.

**Figure 2:**
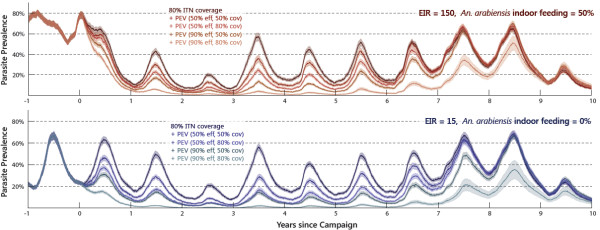
**Effect of mass PEV and ITN distribution on parasite prevalence.** The fraction of individuals with slide-microscopy detectable parasitaemia for one scenario with sustained 80% ITN coverage plus four scenarios layering an additional PEV distribution with 50% (red and blue lines) or 90% (orange and teal lines) efficacy, each at 50% or 80% demographic coverage. Upper panel: Baseline EIR=150, *An.arabiensis* vectors take 50% of feeds indoors. Lower panel: Baseline EIR=15, *An.arabiensis* feeds exclusively outdoors.

Whereas in the previous figure the single mass-vaccination campaign was deployed in isolation, Figure 2 shows the effect of layering the same campaign on top of sustained high ITN coverage levels. The concurrent distribution of ITNs is targeted at 80% of the entire population, with a 90% efficacy for blocking indoor feeds and a 60% killing efficacy. These values are kept constant to model continuing replacement of old nets, including on-going distributions to 80% of newborns. At high transmission intensity (EIR=150) and with 50% indoor *An.arabiensis* feeding (upper panel), the ITNs are responsible for a large reduction in *An.arabiensis* biting and almost complete reduction in the fraction of transmission from the more endophagic *An.gambiaes.s.* and *An.funestus* vectors. While the corresponding PEV-only scenario corresponds to a relatively flat part of the prevalence-to-EIR correlation [53], the ITN-layered scenario is shifted into the steeper region, such that successively higher-coverage campaigns with higher-efficacy vaccines reduce prevalence to near-elimination levels. That is to say, even with an order-of-magnitude higher baseline EIR than the low-transmission, PEV-only scenario from Figure 1, the effectiveness of the mass-vaccination is comparable, on account of the transmission-reduction attributable to ITNs. At low transmission intensity (EIR=15), the ITN intervention alone interrupts transmission within two to three years for the same fraction (50%) of *An.arabiensis* indoor feeds (not shown). However, it is worth noting that even with exclusive outdoor feeding of the *An.arabiensis* species (lower panel), the large reduction in the more ITN-susceptible vectors (especially the dry-season *An.funestus* component) means that the combined interventions can reduce prevalence to near-elimination levels at sufficiently high coverage and efficacy.

**Figure 3:**
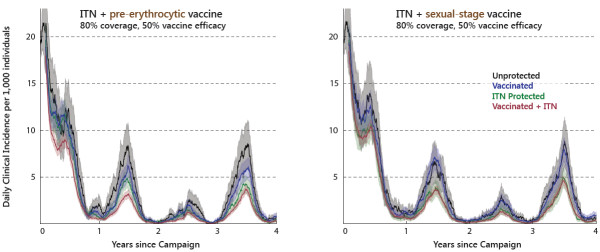
**Clinical cases by intervention status in PEV and sexual-stage vaccine distribution scenarios.** The daily incidence (averaged over a one-month sliding window) of new clinical malaria episodes in over-10 year-olds, grouped according to the protection provided by ITN and vaccine interventions each with 80% demographic coverage: no protection (black), ITN-only (green), vaccine-only (blue), both ITN and vaccine (red). Left panel: Pre-erythrocytic vaccine with 50% efficacy against acquiring infection. Right panel: Sexual-stage vaccine with 50% efficacy against transmission.

While the population-level dynamics of parasite prevalence are similar for pre-erythrocytic and sexual-stage vaccine interventions [39], the different modes of action result in notable differences in disease burden in vaccinated individuals. Figure 3 presents the daily incidence of new clinical cases, specifically malaria-attributable fevers of over 38 degrees Celsius, for individuals over 10 years of age grouped according to intervention status. The act of distributing ITNs to 80% of the population is responsible for a large community-wide reduction in incidence regardless of intervention status. The visible year-to-year variation in clinical cases is a feature driven primarily by the varying levels of annual rainfall in the historical data series, but also to a lesser extent by waning vaccine efficacy. Sleeping under a bed net always provides personal protection on top of the community-level effect on transmission (green line below black in both panels). For the PEV scenario (left panel), individuals have additional personal protection against new clinical cases (blue line below black; red line below green). For the analogous sexual-stage vaccine scenario (right panel), there is no additional personal protection, although for the same vaccine efficacy, the community effect is comparable in magnitude.

**Figure 4:**
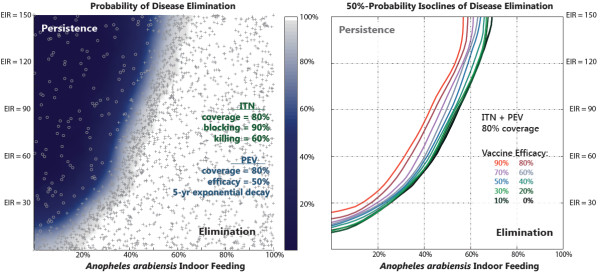
**Regions of disease elimination and persistence after PEV and ITN distribution.** Left panel: The separatrix between the regions of parameter space with disease persistence (dark blue) and local disease elimination (white). Individual simulations are marked either with a cross (for successful elimination) or a circle (disease persistence). ITNs and 50%-efficacy pre-erythrocytic vaccines (PEV) are distributed independently at 80% coverage. Right panel: The location of the separatrix (50% probability isocline) between regions of parameter space with disease persistence for PEV efficacy values ranging from 0% to 90%.

Having explored the characteristic dynamics of large-scale vaccine-plus-ITN campaigns, the following question can be posed: in which geographical and entomological settings can such a campaign locally interrupt malaria transmission? The left panel of Figure 4 maps out the parameter-space regions of disease persistence and elimination as a function of baseline annual EIR and of *An.arabiensis* indoor feeding fraction for a scenario constructed as before with ITNs and PEVs (50% efficacy) independently distributed to 80% of the population. The outcome of each simulation is represented by a grey cross (elimination) or circle (persistence). A novel Separatrix Algorithm was utilized to iteratively infer the probability of disease elimination at any point in phase space based on previous simulations, and then to commission new simulations with parameters most likely to resolve the region of interest that separates persistence from elimination (Klein DJ, Baym M, Eckhoff P. submitted: 2012). The inference part of the algorithm employs a variation on binary kernel regression to calculate probability distributions of ‘success’ (i.e. whether zero infected individuals remain a decade following the campaign intervention) based on the outcomes of all completed simulations. The selection of subsequent simulations in parameter space utilizes a Bayesian design-of-experiments methodology that places samples to maximize the expected information gain (in this case, on the location of the 50% probability isocline in parameter space) as measured by a Kullback-Liebler metric. Disease elimination is achieved at low baseline transmission or high indoor-feeding where ITNs are most effective. The upper-left corner of persistent disease represents settings with high transmission from vectors largely unaffected by ITNs.

The right panel of Figure 4 shows the marginal impact of the additional vaccine intervention and how it depends on vaccine efficacy ranging from 0% to 90%. Each isocline corresponds to the location of the 50% elimination probability isocline inferred as in the left panel. For example, moving along a vertical slice at 50% *An.arabiensis* indoor-feeding, a 50% effective vaccine increases the baseline transmission intensity where elimination is achievable from an EIR of 75 to an EIR of 80. However, a 90% effective vaccine would allow disease elimination for a pre-intervention baseline transmission up to an EIR of 110 when combined with a high-coverage ITN campaign. Note that this range of intermediate EIR values corresponds to relatively large swaths of sub-Saharan Africa as estimated by the Malaria Atlas Project [54]. Alternatively, consider a horizontal slice at a baseline EIR of 20. A 50% effective vaccine reduces the lower limit of the elimination regime from an indoor-feeding fraction of about 18% down to 10%; a 90% effective vaccine ensures that elimination is achieved even for exclusively outdoor-feeding *An.arabiensis*. The ITN campaign completely stops transmission due to the other species, and the vaccine efficacy is high enough to drive residual transmission into stochastic fadeout.

The very significant reduction in transmission (i.e. local elimination) that is shown here for 80% ITN coverage at high endophily is comparable to previous modelling work that has shown dramatic reductions at high population-wide coverage [55]. Similar large-scale reductions in transmission have also been noted in village-scale trials with mass ITN coverage where the dominant vectors were highly endophilic [56,57]. However, several important confounding factors should be noted before attempting to relate these simulations to operational discussions of disease elimination. First, distributed bed nets do not necessarily translate to protected individuals – they may fall into disrepair; they may not cover all residents in a covered household; potentially-protected individuals may not use them every night. The latter effect is especially notable, as individual usage is typically substantially lower than household ownership [1,58-60]. As such, it is important to clarify that the simulations in this paper correspond to consistent ITN *usage* in 80% of simulated *individuals*. Second, the sustained efficacy of ITNs relies on the persistence of susceptible feeding behaviour in the mosquito. While in many cases the indoor fraction of human exposure remains high [61,62], in some settings significant shifts in feeding time and location have been observed [8]. Finally, while the present work focuses on single-village dynamics, the interruption of transmission at larger spatial scales is likely to be significantly more challenging, due to heterogeneity in transmission intensity, spatial structure in ITN coverage [63], and re-introduction from human migration [64,65]. While the present analysis can show generically the magnitude of the potential added-value from layering pre-erythrocytic vaccines on top of mass-distributed ITNs, it will be valuable to address the above considerations in the future in a site-specific fashion.

### Clinical incident reduction with child-targeted campaign

Because of the personal protection provided by even a partially protective pre-erythrocytic vaccine, there are several reasons why it is natural to consider a targeted intervention to minimize cases in young children. This group bears most of the current burden of disease [1], and children have less acquired immunity than older individuals in the same geographic settings [66,67]. Furthermore, EPI represents the most logical existing vaccination infrastructure within which to deploy a new vaccine. To this end, scenarios are constructed that represent an “EPI-plus” rollout of a pre-erythrocytic vaccine (50% efficacy and 5-year decay profile) and a potential future vaccine (90% efficacy) as part of a one-time mass vaccination of under-5 year olds followed by vaccination of new births over the following decade. Both the initial campaign and the ongoing routine vaccination coverage is 80% of targeted individuals.

**Figure 5:**
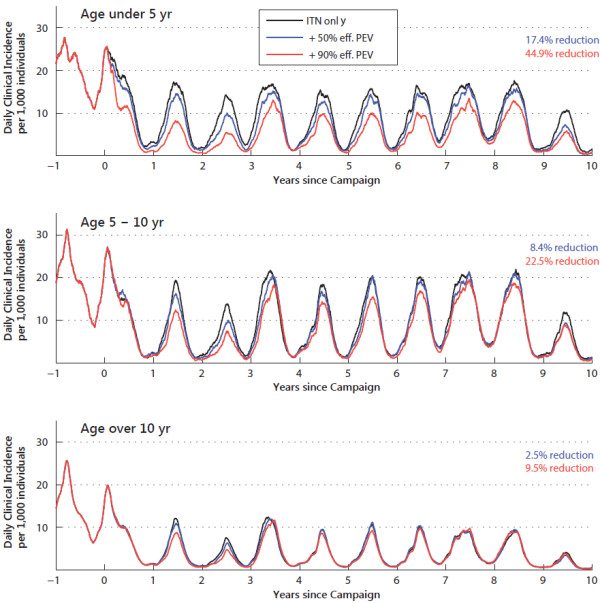
**Reduction in clinical incidence by age after child-targeted PEV campaign.** The daily clinical incidence grouped by age — under 5 years (upper panel), 5 to 10 years (middle panel), over 10 years (lower panel) — in a scenario with 40% population-wide ITN coverage (black lines) compared to scenarios that additionally include a mass-vaccination of under-5 year-olds followed by routine vaccination of newborns with a 50% (blue lines) or 90% (red lines) efficacy PEV at 80% coverage. Daily incidence rates are presented as the running average over a one-month window.

Figure 5 shows the clinical incidence in three age groups (under-5, 5-to-10, and over-10) for a scenario with baseline EIR of 100, exclusive indoor-feeding of the *An.arabiensis* vector, and 40% population coverage of ITNs with the previous set of parameters. Compared to the counterfactual of only bed nets (black line), the figure quantifies the direct benefit of 17.4% (44.9%) fewer cases after year-1 in the under-5 cohort that is targeted with the 50% (90%) efficacy vaccine. Because of the waning vaccine efficacy, the 5-to-10 year-old group experiences a smaller 8.5% (22.5%) reduction in cases. The reduction in cases is more pronounced in the earlier years as the older mass-vaccinated children age into this category. Despite the fact that the over-10 cohort is completely unvaccinated (at least for the first five years until the mass-vaccinated cohort ages in), a small 2.5% (9.5%) reduction is still observed in this age group that is a result of the community effect from fewer infected young children. Note that the community effect on the older age group is quite sensitive to assumptions on what fraction of human-to-vector transmissions are from children and being reduced by the fewer infections in that vaccinated group [68].

**Figure 6:**
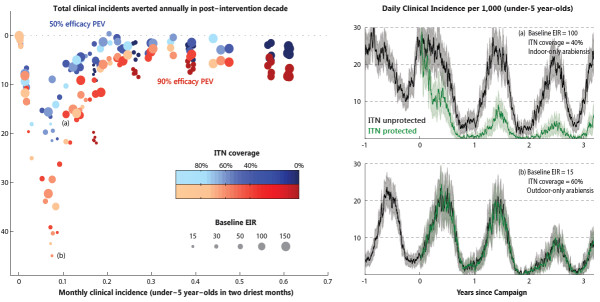
**Summary of clinical cases averted with addition of child-targeted PEV campaign.** Left panel: Summary of the average clinical cases averted in an EPI-like rollout of both an RTS,S-like vaccine (50% efficacy, 5-year exponential decay) and a potential future vaccine (90% efficacy). Incidence reductions are compared to ITN-only scenarios for a parameter sweep over annual baseline EIR, *An.arabiensis* indoor-feeding fraction, and population-wide ITN coverage. The horizontal axis, the monthly under-5 clinical incidence in the two driest months, is a measure of the difficulty to interrupt transmission. The markers indicated by (a) and (b) correspond to the entomological and ITN settings of the subpanels shown on the right. Right panels: Daily clinical incidence of under-5 year-olds with (green) and without (black) ITN protection for the no-vaccine simulations with (a) baseline EIR=100, exclusive indoor-feeding *An.arabiensis*, 40% ITN coverage and (b) baseline EIR=15, exclusive outdoor-feeding *An.arabiensis*, 60% ITN coverage. Daily incidence rates are presented as the running average over a one-week sliding window.

Using the metric of clinical cases averted compared to the ITN-only scenario, different settings are constructed to understand where a vaccine rollout would be most beneficial. To this end, 10 stochastic simulations were run for all combinations of the following three parameters: annual baseline EIR (15, 30, 50, 100, 150), *An.arabiensis* indoor-feeding fraction (0%, 20%, 40%, 60%, 80%, 100%), and population-wide ITN coverage level (0%, 40%, 60%, 80%). The left panel of Figure 6 presents a parameter-sweep summary of the average annual number of clinical cases averted by an EPI-like rollout of either a first-generation pre-erythrocytic vaccine (50% efficacy, bluer symbols) or a potential future vaccine (90% efficacy, redder symbols). In order to calculate the reduction per person-year, one would divide the total reduction in annual incidents by the average population in the post-intervention period, roughly 1,780 individuals in all simulations. The horizontal axis, the monthly under-5 clinical incidence in the two driest months, is a measure of the difficulty to interrupt transmission. It was found to be more predictive than other experimentally measurable quantities like parasite prevalence or annually averaged clinical incidence. The total number of annual clinical cases in the no-vaccine scenarios ranged from less than one on average to more than 160 for the various parameter settings.

At the highest residual transmission levels (EIR=150 with 0% ITN coverage), despite protection from 50% or even 90% of infectious bites, most individuals still spend the majority of the year infected with one or more infections (see Figure 1). It can be easily understood, then, why the total number of clinical cases in a year is only slightly reduced as even vaccinated individuals inevitably become infected. However, the fractional reduction in incidence accelerates at lower residual transmission (i.e. higher ITN coverage or lower baseline EIR as in Figure 2). This transition is also characterized by significant numbers of cases averted in unvaccinated individuals. At very low residual incidence, there are diminishing marginal benefits from vaccinating a whole population to avert the last few cases. The exception to this trend is the realization of continuing dividends upon achieving local elimination. If vaccination could be targeted at identifiable hot-spots [69,70] the cost-effectiveness of the campaign would be extended to lower transmission levels.

It is instructive to look more closely at why the accelerated reduction in clinical cases is more correlated with the level of dry-season transmission than the annually averaged parasite prevalence. The right panels of Figure 6 compare two parameter combinations, for which the annually averaged parasite prevalence in the ITN-only baseline scenario is around 28% and the annual number of clinical cases is around 80. The two scenarios in the right panels correspond to the no-vaccine baselines for the points indicated by (a) and (b) in the left panel. For the first set of parameters (baseline EIR = 100, exclusive indoor-feeding *An.arabiensis*, 40% ITN coverage) shown in the upper-right panel, all vectors are very susceptible to bed nets. This is evident in the pronounced separation in incidence between protected and unprotected individuals. The latter group is responsible for maintaining the moderate level of dry-season transmission. However, for the second set of parameters (baseline EIR = 15, exclusive outdoor-feeding *An.arabiensis*, 60% ITN coverage) shown in the lower-right panel, the *An.arabiensis* component is unaffected by ITNs while the components of transmission due to *An.funestus* and *An.gambiae s.s.* are essentially wiped out. This situation is evident in the homogeneous risk of protected and unprotected individuals to the remaining transmission that is almost exclusively exophagic. The very low transmission during the dry season makes the system sensitive to a relatively modest vaccine rollout. For these reasons, the latter scenario results in 46 cases averted annually, compared to 16 cases averted for the first set of parameters (see the 90% PEV efficacy points indicated in left panel of Figure 6).

At this point, it is worthwhile to note that the predictions of previous modelling efforts are largely consistent with what has been presented in the this paper. It has been noted previously that pre-erythrocytic vaccines should be more effective when deployed in low-transmission environments [34] and more cost-effective when combined with transmission-blocking elements [71]. Similarly, it has been shown in an individual-participant pooled analysis of phase II RTS,S data that vaccine efficacy against clinical episodes drops quickly from 60% at low transmission to 4% at high transmisison [72]. While substantial positive effects of mass-vaccination near the elimination threshold (EIR=2) have been predicted, at higher transmission (EIR=20) EPI should be more cost-effective, especially if it can be targeted at low-transmission areas [36]. Finally, heterogeneity in biting exposure (as e.g. in Figure 6) has also been shown to affect the apparent efficacy of vaccine trial data if the effect is ignored [20].

## Conclusions

Towards the long-term eradication of malaria, the history of other vector-borne diseases, such as yellow fever, suggests that an effective, long-lasting vaccine will be an invaluable tool [26]. Looking at the current generation of vaccines, a goal of global eradication is certainly daunting. However, pushing to higher efficacy (as shown in Figure 4) provides a potentially complementary way of ‘shrinking the map’ [73] of malaria endemic regions beyond what is achievable with high sustained ITN coverage — especially in areas with plentiful exophagic vectors. A few features of the presently constructed mass-vaccination campaign will warrant future studies. In order to maximize the waning vaccine effectiveness at transmission chokepoints, it would appear preferable to engage in mass-vaccinations at least a year following the initial drop in incidence from ramping up ITN coverage. Furthermore, investigations including boosting doses and newborn vaccination would be expected to benefit from sustaining high efficacy over several years before elimination. In this study, vaccine doses and ITNs have been distributed independently. However, while this is an assumption that may be justified under certain circumstances, the modes of distribution may be such that the interventions are more correlated (e.g. ITN distributions at ante-natal clinics [74] or accompanying the vaccination campaign [75]). Finally, the potential to combine pre-erythrocytic and sexual-stage vaccines in a single dosing schedule would likewise provide complementary increased impact.

A broad survey of how a ‘catch-up + EPI’ rollout of an RTS,S-like vaccine can effect a reduction in clinical disease cases has been presented. Over the many scenarios considered, a rough scaling emerges in the magnitude of the clinical case reduction versus the dry-season incidence, revealing subtle thresholds that depend on heterogeneous vector behaviour and intervention coverage. Figure 6 reminds us that for the *same average* transmission levels, homogeneous populations (e.g. the dark-coloured 0% ITN coverage points) are an easier target than populations with heterogeneous baseline intervention coverage. The unprotected sub-population represents a pocket of more persistent transmission, and the protected sub-population already has some potential infections blocked by ITNs, which need not be perfectly complementary to the addition of vaccines. Especially relevant to the question of where vaccines would have the most impact, it has been shown that regions with similar parasite prevalence levels can have significantly different responses to vaccine interventions. One characteristic pattern is where ITN coverage is low and vector behaviour is conducive to ITN control. In this case, a small ramp-up in ITN coverage would not only have a greater effect than introducing a vaccination campaign, but would also make a potential vaccination campaign much more effective. On the other hand, there are settings with negligible marginal benefit of additional ITNs, since only a strongly exophagic residual vector population remains. Especially when the ITN-susceptible vectors were responsible for a large fraction of dry-season transmission (as for the Namawala ecology), this type of setting makes a good candidate for introducing vaccines.

In this paper, ITNs and vaccines have been compared in their combined effectiveness at preventing clinical cases. That is to say, at 80% coverage of the 218 children under age 5 in year-0 and the 62.7 new births per year in the following decade, 680 vaccinations are being dispensed to prevent up to 200 or 450 clinical cases with a 50% or 90% effective vaccine, respectively. These and related models and simulations can thus provide a bridge from vaccine efficacies measured in single-patient challenge studies to the efficaciousness at preventing clinical disease in community rollouts in different settings. Meriting future efforts will be an extension of this study to compare to a metric that is weighted even more to the younger ages who disproportionately experience the most severe symptoms and deaths [1], and to judge the cost of potential vaccine interventions not simply against the absence of additional benefit from more ITNs, but also against other interventions that can provide personal protection against outdoor feeding vectors [76-78].

## Abbreviations

ACT: Artemisinin combination therapy; CSP: Circumsporozoite protein; DTK: Disease transmission kernel; EIR: Entomological inoculation rate; EMOD: Epidemiological modelling; EPI: Expanded programme on immunization; IRBC: Infected red-blood cell; IRS: Indoor residual spraying; ITN: Insecticide-treated net; MSP: Merozoite surface protein; PEV: Pre-erythrocytic vaccine; PfEMP: Plasmodium falciparum erythrocyte membrane protein

## Competing interests

The authors declare that they have no competing interests.

## Authors’ contributions

EW commissioned the simulations, analyzed the results, and drafted the document. PE designed and constructed the malaria model. Both authors have read and approved the final manuscript.
